# PDGFR and IGF-1R Inhibitors Induce a G2/M Arrest and Subsequent Cell Death in Human Glioblastoma Cell Lines

**DOI:** 10.3390/cells7090131

**Published:** 2018-09-06

**Authors:** Estefania Carrasco-Garcia, Isabel Martinez-Lacaci, Leticia Mayor-López, Elena Tristante, Mar Carballo-Santana, Pilar García-Morales, Maria Paz Ventero Martin, Maria Fuentes-Baile, Álvaro Rodriguez-Lescure, Miguel Saceda

**Affiliations:** 1 Instituto de Biología Molecular y Celular, Universidad Miguel Hernández, 03202 Alicante, Spain; carras.estefania@gmail.com (E.C.-G.); imlacaci@umh.es (I.M.-L.); pgarcia@umh.es (P.G.-M.); 2 División de Neurooncología, Instituto Biodonostia, 20014 San Sebastián (Guipuzkoa), Spain; 3 Unidad AECC de Investigación Traslacional en Cáncer, Hospital Clínico Universitario Virgen de la Arrixaca, 30120 Murcia, Spain; letiml27@gmail.com (L.M.-L.); elenatristante86@gmail.com (E.T.); marcarballosantana@gmail.com (M.C.-S.); 4 Unidad de Investigación ISABIAL—FISABIO, Hospital General Universitario de Alicante, 03010 Alicante, Spain; maripazvm@gmail.com (M.P.V.M.); mariafuentesbaile@gmail.com (M.F.-B.); 5 Servicio de Oncología del Hospital General Universitario de Elche, 03203 Alicante, Spain; onkopinha@gmail.com; 6 Unidad de Investigación, Fundación para el Fomento de la Investigación Sanitaria y Biomédica de la Comunidad Valenciana (FISABIO), Hospital General Universitario de Elche, 03203 Alicante, Spain

**Keywords:** glioblastoma, PDFGR, IGF-1R, cell death

## Abstract

Glioblastomas are highly resistant to radiation and chemotherapy. Currently, there are no effective therapies for this type of tumor. Signaling mechanisms initiated by PDGFR and IGF-1R are important in glioblastoma, and inhibition of the signal transduction pathways initiated by these receptors could be a useful alternative strategy for glioblastoma treatment. We have studied the effects of the PDGFR inhibitor JNJ-10198409 (JNJ) and the IGF-1R inhibitor picropodophyllin (PPP) in glioblastoma cell lines as well as in primary cultures derived from patients affected by this type of tumor. JNJ and PPP treatment blocked PDGFR and IGF-1R signaling respectively and reduced Akt and Erk 1/2 phosphorylation. Both inhibitors diminished cell proliferation, inducing a G2/M block of the cell cycle. Cell death induced by JNJ was caspase-dependent, Annexin-V positive and caused PARP cleavage, especially in T98 cells, suggesting an apoptotic mechanism. However, cell death induced by PPP was not completely inhibited by caspase inhibitors in all cell lines apart from LN-229 cells, indicating a caspase-independent mechanism. Several inhibitors targeted against different cell death pathways could not block this caspase-independent component, which may be a non-programmed necrotic mechanism. Apoptotic arrays performed in T98 and LN-229 cells upon JNJ and PPP treatment revealed that procaspase 3 levels were augmented by both drugs in T98 cells and only by JNJ in LN229-cells. Furthermore, XIAP and survivin levels were much higher in LN-229 cells than in T98 cells, revealing that LN-229 cells are more susceptible to undergo caspase-independent cell death mechanisms. JNJ and PPP combination was more effective than each treatment alone.

## 1. Introduction

Malignant gliomas are cancers found frequently in the central nervous system. Among these tumors, the most common subtype, glioblastoma multiforme (GBM), is characterized by being very aggressive and difficult to treat [1]. Glioblastomas are very heterogeneous, have a high proliferation rate and are resistant to chemotherapy and radiation. Nowadays, therapy is based mainly on surgery, radiation and chemotherapy [2]. In spite of the big advances in clinical and translational research, there are no effective therapies for glioblastoma. The first line chemotherapeutic treatment at present is the alkylating agent temozolomide [3]. However, there is a great need for better treatments. Transmembrane tyrosine kinase receptors play an important role in cancer. Upon receptor activation, a signaling cascade is initiated that results in cell proliferation and cell survival. One therapeutic strategy to fight cancer is to shackle these signaling cascades. In fact, molecules involved in signal transduction pathways are used as targets for the treatment of various tumors, including glioblastoma, and numerous inhibitors of these molecules have already entered the clinic or are undergoing clinical trials. PDGFR signaling is important for the onset and progression of glioblastoma [4,5]. Several multi-targeted kinase inhibitors inhibit not only PDGFR, but also VEGFR, FGFR, Bcr-Abl (c-Abl) and stem cell factor receptor (c-Kit). These include: Imatinib, Sorafenib, Nilotinib and Sunitinib [6]. Clinical trials with several PDGFR inhibitors are currently ongoing, showing promising results [7]. In this report, we have utilized the agent *N*-(3-fluorophenyl)-1,4-dihydro-6,7-dimethoxy-indeno[1,2-c]pyrazol-3-amine (JNJ-10198409) to inhibit PDGFR in GBM cell lines. JNJ-10198409 (JNJ) which competes for the ATP binding site in the PDGFR tyrosine kinase domain and has potent antiproliferative activity [8] that is able to inhibit PDGFR downstream signaling in tumor tissues in vivo [9]. Likewise, Insulin-like growth factor 1 receptor (IGF-1R) signaling is very important for glioblastoma progression and development [10,11,12]. Several small molecule inhibitors targeted against IGF-1R have entered clinical trials, while others are in preclinical phase [6]. In this report, we have utilized picropodophyllin (PPP) to inhibit IGF-1R signaling in GBM. This agent is able to inhibit IGF-1R signaling without affecting the Insulin Receptor (IR), due to its binding to the substrate binding site, instead of to the ATP binding site [13]. As a matter of fact, the high homology between IR and IGF-1R represents a drawback for drug design [14]. The cyclolignan PPP is able to cross the blood-brain barrier and is able to reduce both subcutaneous and intracerebral xenografts [15]. We believe that inhibition of the signal transduction pathways initiated by IGF-1R and PDGFR could be a useful alternative strategy for glioblastoma. In fact, several clinical trials with IGF-1R inhibitors, including PPP [16], are currently ongoing for the treatment of glioblastoma [17].

In this report, we sought to determine the effects and mechanisms of actions of JNJ and PPP in a panel of glioblastoma cell lines and primary cultures derived from patients. Both inhibitors inhibited cell growth, and induced G_2_/M arrest and cell death. However, the cell death mechanisms employed by either JNJ or PPP were different and cooperated with each other. Therefore, PDGFR and IGF-1R signaling inhibition are promising strategies for the treatment of glioblastoma patients.

## 2. Materials and Methods

### 2.1. Reagents 

JNJ-10198409 (JNJ) and Picropodophylin (PPP) were purchased from Calbiochem, Merck Millipore (Nottingham, UK). Propidium iodide, 3-(4,5-Dimethylthiazol-2-yl)-2,5-diphenyltetrazolium bromide (MTT), PDGF-BB, IGF-1, nocodazole, chloroquine, AEBSF, Ucf-101 and salubrinal were purchased from Sigma-Aldrich (St. Louis, MO, USA), 7-Amino-Actinomycin D (7-AAD) was purchased from BD Biosciences (Franklin Lakes, NJ, USA). Z-VAD (OMe)-FMK (ICn), Z-D(OMe)E(Ome)VD(OMe)-FMK (IC3), Ac-IETD-CHO (IC8) and necrostatin-1 were purchased from Calbiochem, Merck Millipore (Darmstadt, Germany). MK-1775 was purchased from Selleckchem (Houston, TX, USA).

### 2.2. Cell Culture

The human glioblastoma cell lines U87-MG (U87), A172, T98G (T98) and LN-229 were obtained from the American Type Culture Collection (Manassas, VA, USA), IMR90 fibroblasts were donated from the Instituto Municipal de Investigaciones Médicas (IMIM) Collection (Barcelona, Spain). Cells were maintained in Dulbecco’s modified Eagle’s medium, 4.5 g/L glucose supplemented with 10% heat-inactivated fetal bovine serum (FBS), 2 mM glutamine, 1 mM sodium pyruvate, 50 U/mL of penicillin and 50 mg/mL streptomycin and incubated at 37 °C in a humidified 5% CO_2_/air atmosphere. Cells from primary cultures derived from patients were maintained in Dulbecco’s modified Eagle’s/F-12 medium supplemented with 10% heat-inactivated fetal bovine serum (FBS), 2 mM glutamine, 1 mM sodium pyruvate, 50 U/mL of penicillin and 50 mg/mL streptomycin and incubated at 37 °C in a humidified 5% CO_2_/air atmosphere. Primary cells were used in the first 10 passages and designated as “HGUE-GB-xx”, xx being a number related to a particular patient in an anonymous manner.

### 2.3. Ethical Statement

Primary glioblastoma cell culture samples were obtained from surgical aspirates after tumor resection was performed on patients at the Hospital General Universitario de Elche, who had already signed an informed consent form (Elche, Alicante, Spain), and provided by the Hospital Biobank, according to institutional human ethic guidelines and approved by the Ethics Committee of the Hospital General Universitario de Elche.

### 2.4. Cell Proliferation Assays

Cells were plated in 96-well flat bottom plates at a density of 2–3 × 10^3^ cells per well, depending on the cell line, and treated 24 h later with vehicle (DMSO) or different doses of PPP or JNJ-10198409 for 72 h in sextuplicate. Subsequently, 0.25 mg/mL of MTT reagent was added in each well and incubated for 3 h at 37 °C in a humidified 5% CO_2_/air atmosphere. After the incubation, the media was removed and 200 μL of DMSO were added to each well to dissolve the formazan product formed. Then, after shaking for 30 min at room temperature, the absorbance was measured at 570 nm in a microplate reader (Anthos 2001 Labtec Instruments GmbH, Anthos, Salzburg, Austria). 

### 2.5. Flow Cytometric Analysis of Cell Cycle Phase Distribution

Cells were plated and treated with 500 nM JNJ or 500 nM PPP for particular times. Cells were trypsinized, washed with PBS, fixed with 75% cold ethanol at −20 °C for at least 1 h, incubated with 0.5% Triton X-100 and 25 µg/mL RNase A in PBS for 30 min at room temperature, stained with 25 ng/mL propidium iodide and incubated for 15 min in the dark to counterstain DNA, and analyzed using an Epics XL flow cytometer (Beckman Coulter Co., Miami, FL, USA).

### 2.6. p-Histone H3 Staining 

Cells were plated and treated with 500 nM JNJ or 500 nM PPP, trypsinized, washed with PBS, fixed with 75% cold ethanol at −20 °C, washed and incubated with 0.25% Triton X-100/PBS (Sigma-Aldrich, St. Louis, MO, USA) for 5 min at 4 °C. The samples were incubated for 2 h at room temperature in the dark with an anti-Histone H3 phosphorylated (Ser 28) antibody conjugated to Alexa Fluor 647 (clone HTA 28), or the isotypic control antibody-Alexa Fluor 647 (clone RTK 2758) containing 1% bovine serum albumin (BSA) (Biolegend Inc., San Diego, CA, USA) in PBS. Samples were washed, incubated with 25 μg/mL RNase A in PBS for 30 min at room temperature, stained with 25 ng/mL propidium iodide and incubated for 15 min in the dark to counterstain DNA, and then analyzed by flow cytometry using a BD FACSCanto™ flow cytometer (Becton Dickinson & Co., Franklin Lakes, NJ, USA). 

### 2.7. Annexin-V Staining 

Cells were plated, treated with JNJ or PPP, harvested, washed with cold PBS, resuspended in a binding buffer containing 10 mm Hepes pH 7.4, 140 mm NaCl and 2.5 nm CaCl_2_. Then, 100 μL (1 × 10^5^ cells) of cells were transferred to new tubes, incubated with 5 μL of fluorescein isothiocynate (FITC) Annexin-V (Biolegend Inc., San Diego, CA, USA) and 5 μL of 7-Amino-Actinomycin D (7-AAD) for 15 min at room temperature in the dark, according to manufacturer’s instructions (Becton Dickinson, Franklin Lakes, NJ, USA) and analyzed by cytometry using a BD FACSCanto™ flow cytometer.

### 2.8. Chromatin Fragmentation 

Cells were plated at a density of 15·× 10^3^ cells/well in 24-well plates (2 cm^2^ of area) and after 24 h, treated with 500 nM JNJ or 500 nM PPP for different times, harvested and washed with PBS. Cells were fixed by incubation with cold methanol for at least 1 h at −20 °C. Then, cells were incubated for 15 min with 3 μg/mL Hoechst 33342 (Molecular Probes, Life Technologies, Grand Island, NY, USA) at room temperature in the dark, visualized under a fluorescence microscope (Nikon Eclipse T2000 U) and photographed using NIS elements software (Nikon, Tokyo, Japan). 

### 2.9. Western Blot Analysis

Cells were plated, treated with 500 nm JNJ or 500 nM PPP for different times and lysed in NP-40 lysis buffer (50 mM Tris-HCl pH 7.4, 1% NP-40, 150 mM NaCl, 5 mM EDTA, 50 mM NaF, 30 mM Na_4_P_2_O_7_, 1 mM Na_3_VO_4_,) with protease inhibitor cocktail (Sigma-Aldrich, St. Louis, MO, USA) for 30 min on ice. After centrifugation at 15,000× *g* for 5 min at 4 °C, the supernatants were collected, and protein concentrations were determined by the Bradford method (Bio-Rad, Richmond, CA, USA). Then, 50 μg of protein from each lysate were resolved by SDS-PAGE, under reducing conditions. Depending on the protein under study, we used 7.5% (for larger proteins), 10% (for medium size proteins) or 12% (for smaller protein) polyacrylamide gels. Subsequently, proteins were transferred to nitrocellulose membranes, using a BioRad system for 1.5 h at 100 V in a buffer containing 25 mm Tris, 192 mm Glycine and 20% methanol and blocked with 5% non-fat dry milk or BSA (depending on the antibody, and according to manufacturer’s recommendations) in TBST (137 mm NaCl, 20 mm Tris pH 7.6, 0.05% Tween) buffer for 1 h. Then, proteins were incubated with primary antibodies for 16 h at 4 °C by gentle rocking using different concentrations (usually 1:500 for phosphoantibodies and 1:1000 for the rest of the antibodies), depending on the antibody against PDGFR-α (sc-431), PDGFR-β (sc-432), phospho-PDGFR-β (Tyr 857) (sc-12907), IGF-1R (sc-731), phospho-tyrosine (sc-508), Akt (sc-8312), cdc2 (sc-54), cyclin B_1_ (sc-594), Plk (sc-55504), phospho-histone H3 (Ser 10) (sc-8656), phospho-IGF-1R (Tyr 1161) (sc-713) from Santa Cruz Biotechnology (Santa Cruz, CA, USA), phospho-Akt (Ser 473) (No. 9271), phospho-Erk 1/2 (Thr 202/Tyr 204) (No. 9101), Erk 1/2 (No. 91002), phospho-cdc2 (Tyr 15) (No. 4539), phospho-cdc2 (Thr161) (No. 9114), PARP (No. 9542) from Cell Signaling Technologies (Beverly, MA, USA), or β-actin (A 2066) (Sigma-Aldrich) and incubated with horseradish peroxidase-linked secondary antibodies (Amersham, GE Healthcare, Buckinghamshire, UK). Proteins were detected by the ECL system (Amersham, GE Healthcare). Densitometric analyses were performed using the Scion Image software, version 4.0.3.2, Scion Corporation (Frederick, MD, USA).

### 2.10. Immunoprecipitation 

Cells were treated, harvested and lysed as above. For the immunoprecipitation of PDGFR-α, 800 μg of protein were incubated with 3 μg of the antibody against PDGFR-α (sc-431, Santa Cruz Biotechnology, Dallas, TX, USA) or a control IgG (Sigma-Aldrich) in a final volume of 1 mL of lysis buffer and rotated for 16 h at 4 °C. Then, 25 μL of protein G-Agarose beads (Pierce Chemical Co., Rockford, IL, USA) were added and rotated for 1 h at 4 °C. Subsequently, beads were washed four times with 0.5 mL of lysis buffer and resuspended in 30 μL of SDS-PAGE sample buffer. Samples were denatured by boiling in a sample buffer at 95 °C for 5 min and then centrifuged (1000× *g* for 5 min). Supernatants were subjected to Western blot analysis using the antibody already mentioned or an antibody against phospho tyrosine (sc-508, Santa Cruz Biotechnology).

### 2.11. Apoptosis Array 

Cells were plated in 145-cm culture plates at a density of 3·× 10^6^ cells/plate and, after 24 h, treated with 500 nM JNJ or 500 nM PPP for 24 h and lysed in NP-40 lysis buffer (1% NP-40, 10% glycerol, 20 mM Tris-HCl pH 8, 137 mM NaCl, 2 mM EDTA, 1mm sodium orthovanadate and proteases inhibitors). Protein concentrations were determined by the Bradford method and the apoptotic proteome profile was determined using the Human Apoptosis Array (ARY009, R&D Systems, Minneapolis, MN, USA), according to the manufacturer’s instructions. Subsequently, 500 μg of each lysate was diluted to 1.5 mL and incubated on the nitrocellulose membranes for 16 h at 4 °C on a rocking platform shaker. Then, membranes were washed three times and then incubated with a cocktail of biotinylated detection antibodies for 1 h at room temperature on a rocking platform shaker. After three washes, membranes were incubated with Stretpavidin-horseradish peroxidase for 30 min on a rocking platform shaker and were exposed to film (Hyperfilm ECL ref. 28906836, Amersham, GE Healthcare). Films were developed using developer and fixer solutions in a Kodak X-OMAT-1000 processor (Eastman Kodak Company, Rochester, NY, USA), according to the manufacturer’s instructions. Densitometric analyses were performed using the Scion Image software, version 4.0.3.2 (Scion Corporation).

### 2.12. Statistical Analysis 

The results are shown as the mean ± standard error of the mean (S.E.M.) of at least three independent experiments. First, a descriptive statistic was performed with the, GraphPad Prism version 5 (GraphPad Software Inc., San Diego CA, USA) calculating the mean and standard deviation for the values. The normality of the data was evaluated by means of the Shapiro-Wilk statistical test, and homoscedasticity was verified using Levene’s statistical test. The comparisons between two groups were carried out by means of the Student’s *t*-test in parametric and homescedastic data sets, and using the U Mann Whitney statistical test for the data, that not comply with the principles of normality and homoscedasticity. Values of *p* < 0.05 or less indicated a significant difference. 

## 3. Results

### 3.1. Expression Levels of PDGFR and IGF-1R in Glioblastoma Cell Lines 

**Figure 1:**
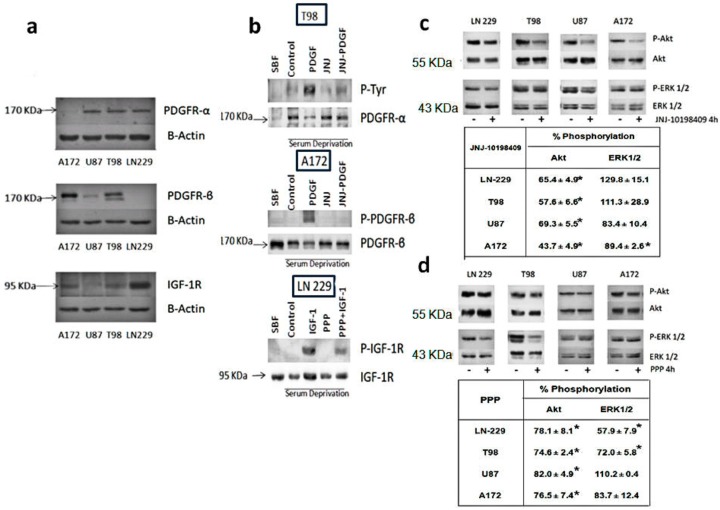
Expression, activation and downstream signaling of PDGFR and IGF-1R. (**a**) PDGFR and IGF-1R expression levels. PDGFR-α, PDGFR-β and IGF-1R levels in GBM cell lines were analyzed by Western blot. β-actin was used as an internal control. (**b**) PDGFR and IGF-1R phosphorylation levels. T98 or A172 cells were grown in 10% FBS-containing media (FBS) and serum-starved for 24 h. Then, cells were non-treated (control), or pre-treated with 500 nM JNJ-10198409 (JNJ) for 30 min and challenged with 100 ng/mL PDGF-BB for an additional 10 min. PDGFR-α was immunoprecipitated and subjected to Western blot using antibodies against p-Tyr and PDGFR-α in T98 cell extracts or antibodies against phospho-PDGFR-β or PDGFR-β in A172 cell extracts. LN-229 cells were grown in 10% FBS-containing media (FBS) and serum-starved for 24 h. Then, cells were non-treated (control), or pre-treated with 500 nM picropodophyllin (PPP) for 30 min and challenged with 100 ng/mL IGF-1 for an additional 10 min. Proteins were extracted and subjected to Western blot using antibodies against phospho-IGF-1R (Tyr 857) or IGF-1R. (**c**,**d**) Effects of JNJ and PPP on Akt and Erk phosphorylation. GBM cell lines were treated (+) or left untreated (−) with 500 nM JNJ (**c**) or 500 nM PPP (**d**) for 4 h. Cell extracts were obtained and analyzed by Western blot using the appropriate antibodies to determine phosphorylation levels. β-actin was used as an internal control. Optical densitometry was done to determine the level of expression of the phosphorylates as well as the total proteins. Phosphorylation levels were determined as the ratio between the phosphorylated form and the total. Tables included in figure (**c**) and (**d**) represent percent of phosphorylated form (mean ± S.E.M.) obtained after four independents experiments. * Means a significative change in phosphorylation, as compared to the phosphorylation levels in control untreated cells, *p* < 0.05.

Western blot analyses were performed in order to determine the protein expression levels of PDGFR-α, PDGFR-β and IGF-1R in the glioblastoma cell lines utilized in our study. PDGFR-α protein levels are moderate in U87, T98 and LN-229 cells and barely detectable in A172 cells. In contrast, A172 cells express high levels of PDGFR-β, followed by T98 cells. PDGFR-β levels are very low in U87 cells and non-detectable in LN-229 cells. IGF-1R protein levels are elevated in LN-229 cells, followed by A172, LN-229 cells and barely detectable in U87 cells (Figure 1a).

### 3.2. Inhibition of Phosphorylation of PDGFR, IGFR-1R and Downstream Pathways

In order to determine whether JNJ was able to inhibit the α and β isoforms of PDGFR, we analyzed the PDGFR phosphorylation status in serum-deprived cells in response to PDGF-BB, which activates both the α and β isoforms, in the presence or absence of the inhibitor. JNJ completely abolished the phosphorylation of PDGFR-β and was able to inhibit to a great extent PDGFR-α phosphorylation (Figure 1b). Likewise, PPP was able to inhibit IGF-1R phosphorylation in cells challenged with IGF-1 (Figure 1b). Akt phosphorylation levels diminished after JNJ treatment in the four GBM cell lines, especially in A172 cells, whereas Erk 1/2 phosphorylation levels decreased only in U87 and A172 cells (Figure 1c). Akt phosphorylation levels also decreased after PPP treatment in all GBM cell lines. However, the Erk 1/2 phosphorylation decrease was more relevant in LN-229 and T98 cells, only moderate in A172 cells and did not occur in U87 cells, where the Akt phosphorylation decrease was also modest (Figure 1d).

### 3.3. JNJ and PPP Inhibit Proliferation of Glioblastoma Cell Lines and Primary Cultures 

**Figure 2:**
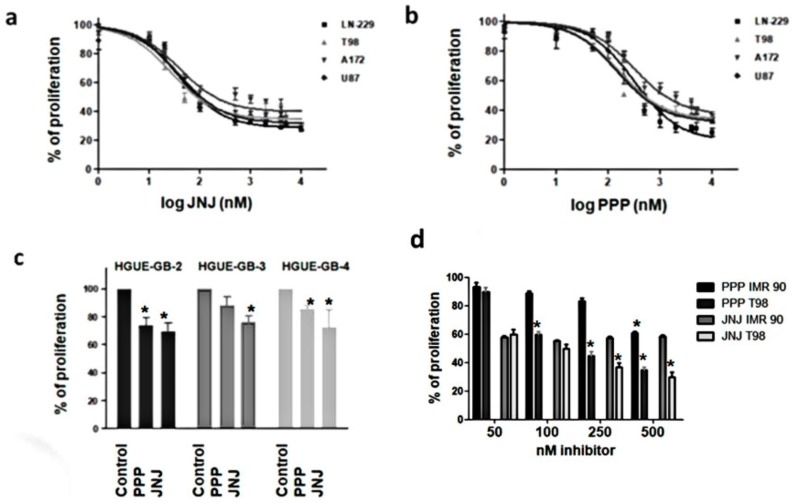
JNJ and PPP effects on GBM cell proliferation. (**a**,**b**) LN-229, T98, A172, and U87 were grown in 96-well plates, treated with DMSO (vehicle) or different concentrations of JNJ (**a**) or PPP (**b**) for 72 h, incubated with MTT, dissolved with DMSO, and cell proliferation rates were determined by colorimetry, as described in Materials and Methods, represented as the average of at least seven independent experiments with six replicates in each experiment data point and referred to as percentage of control. Error bars are the S.E.M. (**c**) Primary cultures derived from tumors were grown, treated with DMSO 500 nm PPP, 500 nm JNJ for 72 h and cell proliferation rates were determined using the MTT method. (**d**) T98 and IMR90 were grown in 96-well plates, treated with DMSO (vehicle) or different concentrations of JNJ or PPP for 72 h, incubated with MTT, dissolved with DMSO, and cell proliferation rates were determined by colorimetry, as described in Materials and Methods. Error bars are the S.E.M. values in (**c**,**d**), represented as the average of three independent experiments with six replicates in each experimental data point. * Means a statistically significative change, as compared to control untreated cells, *p* < 0.05.

JNJ inhibited the proliferation of the four glioblastoma cell lines, with an IC_50_ of 26.1 ± 1.2 nM in T98 cells, 39.0 ± 1.1 nM in LN-229 cells, 40.4 ± 1.1 nM in U87 cells and 44.7 ± 1.2 nM in A172 cells (Figure 2a). PPP was able to inhibit glioblastoma cell proliferation as well, but with a higher IC_50_ of 138.9 ± 1.1 nM in U87 cells, 145.4 ± 1.2 nM in T98 cells, 286.7 ± 1.1 nM in LN-229 cells and 330.0 ± 1.2 nM in A172 (Figure 2b). Therefore, the response of the four cell lines to the inhibitors was very similar. We wanted to extend or studies to primary cultures derived from tumors obtained from surgical aspirates after tumor resection of glioblastoma patients. Again, the effect of JNJ was higher than the effect of PPP on the inhibition of cell growth of primary cultures, named as HGUE-GB-2, HGUE-GB-3 and HGUE-GB-4 (Figure 2c). As a control, we test the effects of PPP and JNJ in a non-tumor cell line, IMR90. In Figure 2d, we compare the effect of PPP and JNJ (50–500 nm) on IMR90 and T98 cell proliferation. Our data show that PPP only had a significative effect on IMR90 proliferation at 500 nm and that even this effect at 500 nm was significative lower than the effect of the same concentration of PPP in the tumoral T98 cell line. In reference to JNJ, both cell lines have similar levels of proliferation inhibition at low JNJ concentrations (50–100 nm). However, at higher concentrations, the tumoral T98 cell line was significantly more affected by JNJ than IMR90 cells. It is important to mention that fibroblasts, such as the IMR90 cells, express PDGFR and that PDGF is a mitogen for this type of cell. Interestingly, in contrast to the GBM cell lines used in this study, cell cycle analysis shows that PPP and JNJ induced a G_2_/M arrest in IMR90 fibroblasts, and were not followed by cell death (data not shown). 

### 3.4. Effects of JNJ and PPP on Glioblastoma Cell Cycle

**Figure 3:**
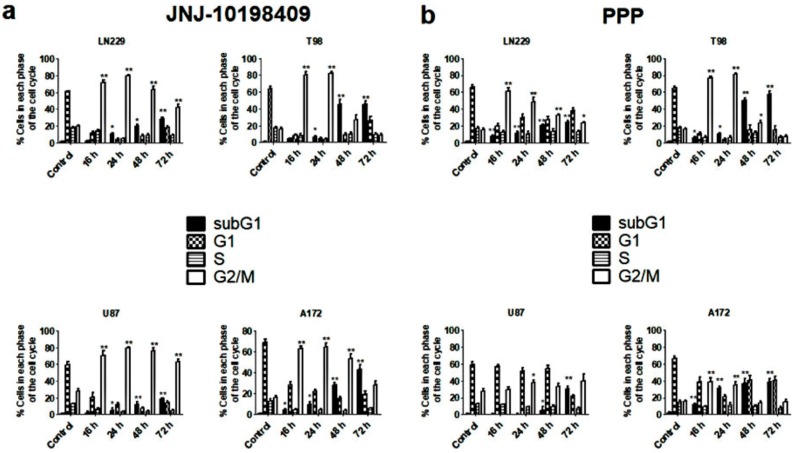
JNJ and PPP effects on cell cycle distribution. GBM cell lines were treated with DMSO, 500 nm JNJ (**a**) or 500 nm PPP (**b**) for 16, 24, 48 and 72 h; and cell cycle distribution of DNA content was determined by flow cytometry, as described in Materials and Methods. The percentage of cells in each phase of the cell cycle is represented as the average of six independent experiments, *n* = 6. Values are presented as the percentage of cell in each phase of the cell cycle. Error bars are the S.E.M. * Means a statistically significative change, as compared to control untreated cells, *p* < 0.05 ** *p* < 0.01.

In order to determine the effects of JNJ and PPP on cell cycle distribution of DNA content, we performed cell cycle analyses after 16, 24, 48 and 72 h and found that both inhibitors produced significant changes on cell cycle profiles. In response to JNJ, the four cell lines accumulated in the G_2_/M phase of the cell cycle within 16 h at the expense of the G_1_ fraction that decreased substantially. Subsequent to the G_2_/M arrest, cells accumulated in the SubG_1_ phase, especially after 48 or 72 h of JNJ treatment. This effect was more evident in T98 cells (Figure 3a). Likewise, glioblastoma cell lines were arrested in the G_2_/M phase, followed by SubG_1_ phase accumulation after PPP treatment (Figure 3b). However, U87 cells were less affected by PPP.

### 3.5. G_2_/M Arrest Induced by JNJ and PPP 

**Figure 4:**
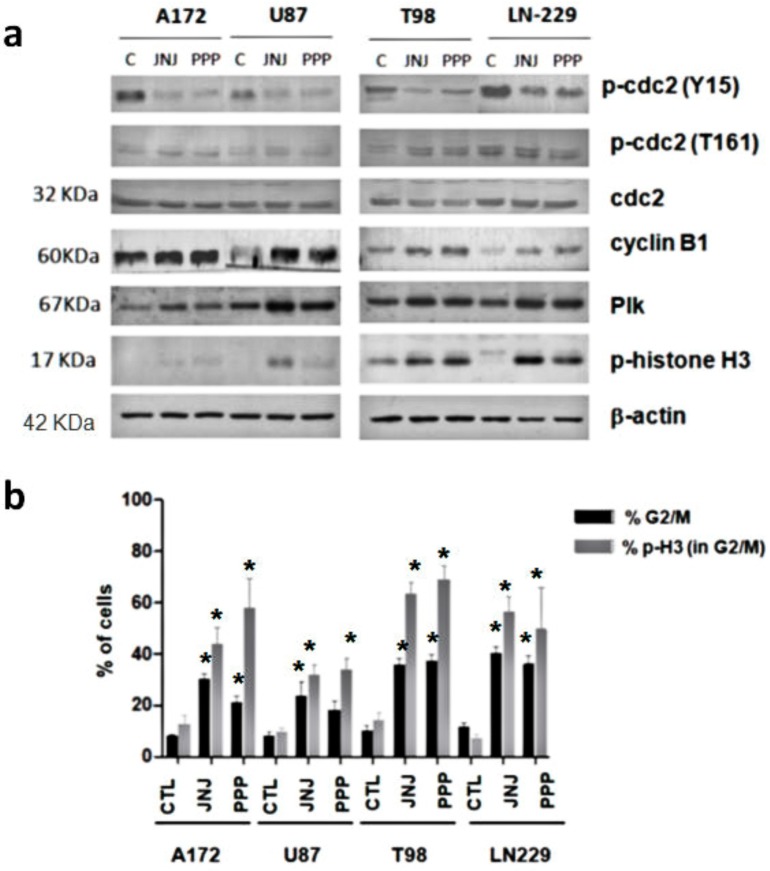
Study of the G_2_/M arrest. (**a**) JNJ and PPP effects on G_2_/M checkpoint proteins. LN-229 cells were treated with DMSO (C), 500 nm JNJ or 500 nm PPP for 16 h and subjected to Western blot analysis using the antibodies indicated. ↕-actin was used as a loading control. (**b**) Induction of mitotic arrest. GBM cell lines were seeded, treated with DMSO (CTL), 500 nm JNJ or 500 PPP for 16 h and stained with phospho-histone H3 antibody and propidium iodide, as described in Materials and Methods. The percentage of cells in the G_2_/M phase (black bars) and the percentage of p-histone H3 positive cells within the G_2_/M phase (grey bars) are represented as the average of four independent experiments. Error bars are the S.E.M. * Mean a statistically significative change, as compared to control untreated cells, *p* < 0.05.

In order to study in further detail the G_2_/M arrest induced by both inhibitors, we analyzed the levels and activation of proteins involved in the G_2_/M checkpoint. The steady-state levels of cdc2 protein were unaffected. However, the treatment with JNJ or PPP caused a decrease in the phosphorylation levels of cdc2 (Tyr 15) and a slight increase in the phosphorylation levels of cdc2 (Thr 161), which indicates M phase entry. Furthermore, the steady-state levels of cyclin B_1_ protein were induced, suggesting that cells were not able to exit from mitosis and accumulated in this phase of the cell cycle (Figure 4a). In addition, Polo-like kinase (Plk) protein levels, which regulate mitotic spindle function [18], were elevated after 16 hours of treatment with either JNJ or PPP in the four glioblastoma cell lines (Figure 4a). Furthermore, histone H3 phosphorylation levels were also elevated, as revealed by Western blot analyses (Figure 4a) and flow cytometry, especially in T98 and LN-229 cells (Figure 4b). Flow cytometric analyses with DNA content and phosphorylated-histone H3 to distinguish between G_2_ and mitotic cells were conducted. As the extent of histone H3 is involved in chromosome condensation and is considered a marker of mitosis [19,20,21], we can observe that in general, there are a higher percentage of cells in mitosis, considering the G_2_/M-arrested cell population, after PPP treatment than after JNJ treatment. In addition, IGF-1R inhibition by PPP caused nuclei fragmentation and aggregation and micronuclei formation, characteristic of mitotic catastrophe or cell death due to mitotic arrest (Figure S1). 

### 3.6. Are Caspases Involved in JNJ- or PPP-Induced Programmed Cell Death? 

**Figure 5:**
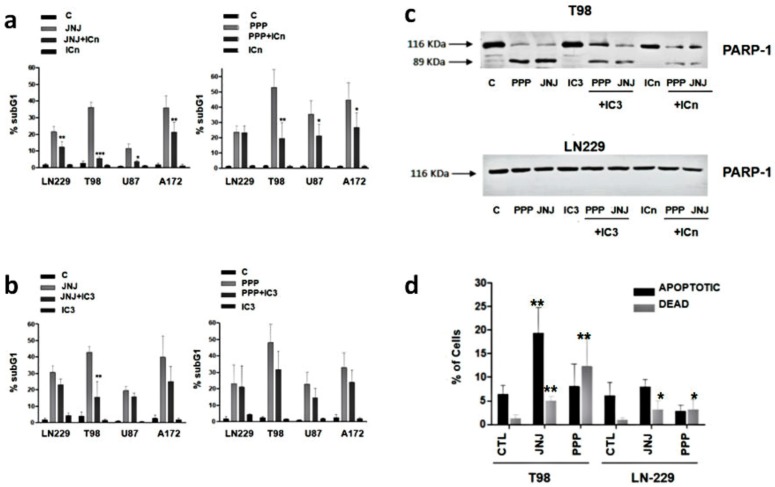
Role of caspases in JNJ and PPP-induced cell death. (**a**) Effects of the pan caspase inhibitor. GBM cell lines were left untreated (**c**) or treated with 500 nM JNJ (left panel), 500 nM PPP (right panel), 25 μM of the general caspase inhibitor Z-VAD (OMe)-FMK (ICn), or a combination of either JNJ and ICn and PPP and ICn for 72 h, after a 30 min pretreatment period with only ICn. Then, cells were subjected to cell cycle analyses, as described in Materials and Methods. The percentage of cells in the SubG_1_ phase is represented as the average of 6 (*n* = 6) independent experiments. Difference between means was statistically significant * (*p* < 0.05), ** (*p* < 0.01), *** (*p* < 0.001). (**b**) Effects of the specific caspase-3 inhibitor. GBM cell lines were left untreated (C) or treated with 500 nM JNJ (left panel), 500 nM PPP (right) panel), 5 nM of the specific caspase-3 inhibitor Z-D(OMe)E(Ome)VD(OMe)-FMK (IC3), or a combination of either JNJ and IC3 and PPP and IC3 for 72 h, after a 30 min pretreatment period with only IC3. Then, cells were subjected to cell cycle analyses, as described in Materials and Methods. The percentage of cells in the SubG_1_ phase is represented as the average of three independent experiments. Difference between means was statistically significant * (*p* < 0.05), ** (*p* < 0.01). (**c**) PARP processing. T98 and LN-229 cells were left untreated (**c**) or treated with 500 nM JNJ or 500 nM PPP, in the presence or absence or 25 μM Z-VAD (OMe)-FMK (ICn), or 5 μM Z-D(OMe)E(Ome)VD(OMe)-FMK (IC3). Cells were subjected to Western blot analysis using a PARP antibody. (**d**) Annexin-V staining. T98 and LN-229 cell lines were treated with DMSO (CTL), 500 nM JNJ or 500 nM PPP for 16 h and stained with FITC Annexin-V, as described in Materials and Methods. The percentage of Annexin-V positive/7-AAD negative (apoptotic) cells and Annexin-V negative/7-AAD positive (dead) cells is represented as the average of three separate experiments. Error bars are the S.E.M.

We sought to determine the mechanism of cell death due to JNJ or PPP treatment. As caspases are involved in the classical route of apoptotic death, we treated glioblastoma cells with caspase inhibitors in order to block the JNJ or PPP-dependent cell death. The pan-caspase inhibitor Z-VAD (OMe)-FMK (ICn) was able to significantly reduce the percentage of cell death induced by JNJ in the four GBM cell lines upon 72 h of treatment (Figure 5a). However, this inhibitor was able to reduce only partially the amount of cell death due to PPP in T98, U87 and A172 cells, and had no effect in LN-229 cells. The specific caspase 3 inhibitor Z-D(OMe)E(Ome)VD(OMe)-FMK (IC3) was able to inhibit JNJ-dependent cell death mainly in T98 cells. PPP-dependent cell death was abolished with IC3 in T98, U87 and A172 cells to a small extent and like ICn, had no effect in LN-229 cells (Figure 5b). Therefore, caspases are involved in JNJ-dependent cell death, but only to a certain extent in PPP-dependent cell death. As PARP is fragmented by caspases in cell death mechanisms, we analyzed PARP cleavage by Western blot and found that both JNJ and PPP induced cleavage of PARP that could be prevented especially by ICn and to a lesser extent by IC3 in T98 cells (Figure 5c). However, PARP cleavage did not occur in LN-229 after 24 h of JNJ or PPP treatment (Figure 5c). 

### 3.7. Is Apoptotic Cell Death Induced by JNJ or PPP? 

An early apoptotic event is phosphatidyl serine exposure to the outer leaflet of the cell membrane. In order to determine whether the cell death induced by JNJ or PPP was a classical apoptotic mechanism, cells were stained for membrane-exposed phosphatidylserine utilizing Annexin-V assays. We found that the percentage of Annexin-V positive/7-AAD negative cells, indicative of early apoptosis, increased after JNJ treatment, especially in T98 cells and to a lesser extent in LN-229 cells, where the amount of dead cells was also smaller. Interestingly, we found that the ratio of Annexin-V negative/7-AAD positive cells, indicative of dead cells that have lost membrane integrity, increased after PPP treatment, suggesting that JNJ induces an apoptotic form of cell death, whereas PPP does not (Figure 5d).

### 3.8. Alternative Mechanisms of Programmed Cell Death

**Figure 6:**
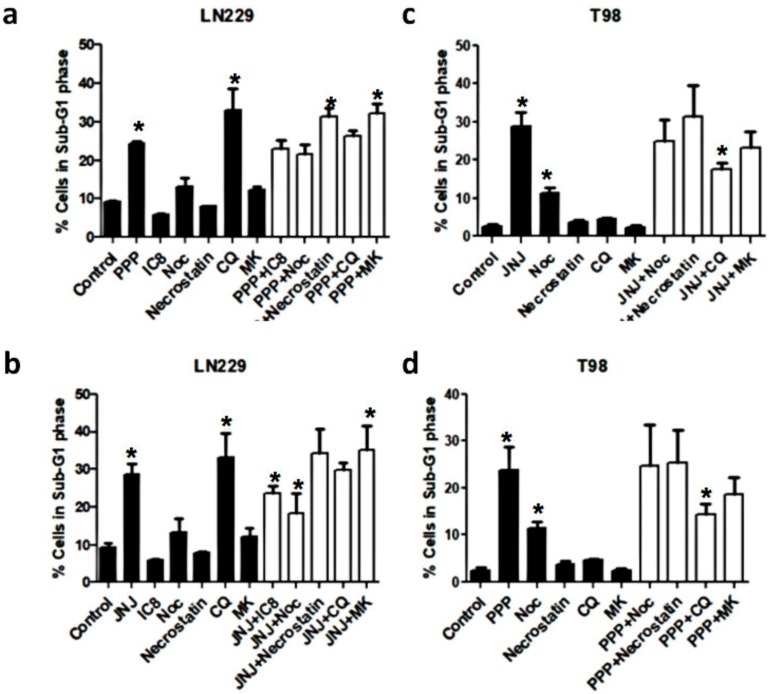
Role of other inhibitors in JNJ and PPP-induced cell death. (**a**,**b**) LN-229 cells were treated with DMSO (Control), 500 nM JNJ or 500 nM PPP in the presence or absence of 25 μM of the specific caspase-8 inhibitor Ac-IETD-CHO (IC8), 1 μg/mL Nocodazole (Noc), 10 μM Necrostatin-1, 20 μM chloroquine (CQ), or 100 nM MK-1775 for 24 h, after a 30 min pretreatment with these inhibitors. (**c**,**d**) T98 cells were treated with DMSO (Control), 500 nM JNJ or 500 nM PPP in the presence or absence of 1 μg/mL Nocodazole (Noc), 10 μM Necrostatin-1, 20 μM chloroquine (CQ), or 100 nM MK-1775 for 24 h, after a 30 min pretreatment with these inhibitors. Then, cells were subjected to cell cycle analyses, as described in Materials and Methods. The percentage of cells in the SubG_1_ phase is represented as the average of more than three independent experiments. Black bars represent treatments as single agents. White bars represent combination of treatments. Error bars are the S.E.M. (*n* = 16; control, PPP or JNJ treatment are the conditions present in 16 independent experiments, all the other conditions were assayed in at least six of the total 16 experiments). * Means a statistically significative change in PPP or JNJ treated cells (white filled bars), as compared to the control untreated cells (black filled bars), *p* < 0.05.

As PPP induces a non-typical class of programmed cell death which is more prevalent in LN-229 cells, we have employed a variety of inhibitors in order to identify an alternative cell death mechanism (Figure 6). In order to investigate whether the extrinsic route was involved, we employed the caspase 8 inhibitor Ac-IETD-CHO (IC8) [22]. However, this inhibitor did not abolish the cell death induced by either PPP or JNJ in LN-229 cells after 24 h of treatment. In addition, we wanted to investigate whether agents that affect the G_2_/M phase checkpoint could inhibit the cell death provoked by PPP or JNJ. The microtubule inhibitor nocodazole had some partial effect when used as a single agent, especially in LN-229 cells. However, the percentage of cells in the SubG_1_ phase after co-treatment with nocodazole and either PPP or JNJ was basically the same as the percentage of dead cells induced by PPP or JNJ as single agents. The wee-1 inhibitor MK-1775 [23] also had some partial effect when used as a single agent in LN-229 cells, but it was unable to rescue cell death either after PPP or JNJ. The necropotosis inhibitor necrostatin-1 [24] could not rescue cells from cell death in any case, indicating that neither PPP nor JNJ induced necroptosis. In contrast, the autophagy inhibitor chloroquine [25] was able to induce cell death when used as a single agent in LN-229 cells, but not in T98 cells. Interestingly, chloroquine partially diminished cell death induced by either PPP or JNJ in T98 cells. Other agents such as the serine protease HTRA2/Omi [26] inhibitor Ucf-101 [27], serine proteases inhibitor 4-(2-Aminoethyl)-benzenesulfonyl fluoride (AEBSF) [28,29], and eIF2α inhibitor salubrinal were utilized [30,31] (Figure S2) in LN-229 cells, but were unable to abolished PPP-induced cell death.

### 3.9. Molecular Characterization of JNJ- and PPP-Induced Cell Death 

**Figure 7:**
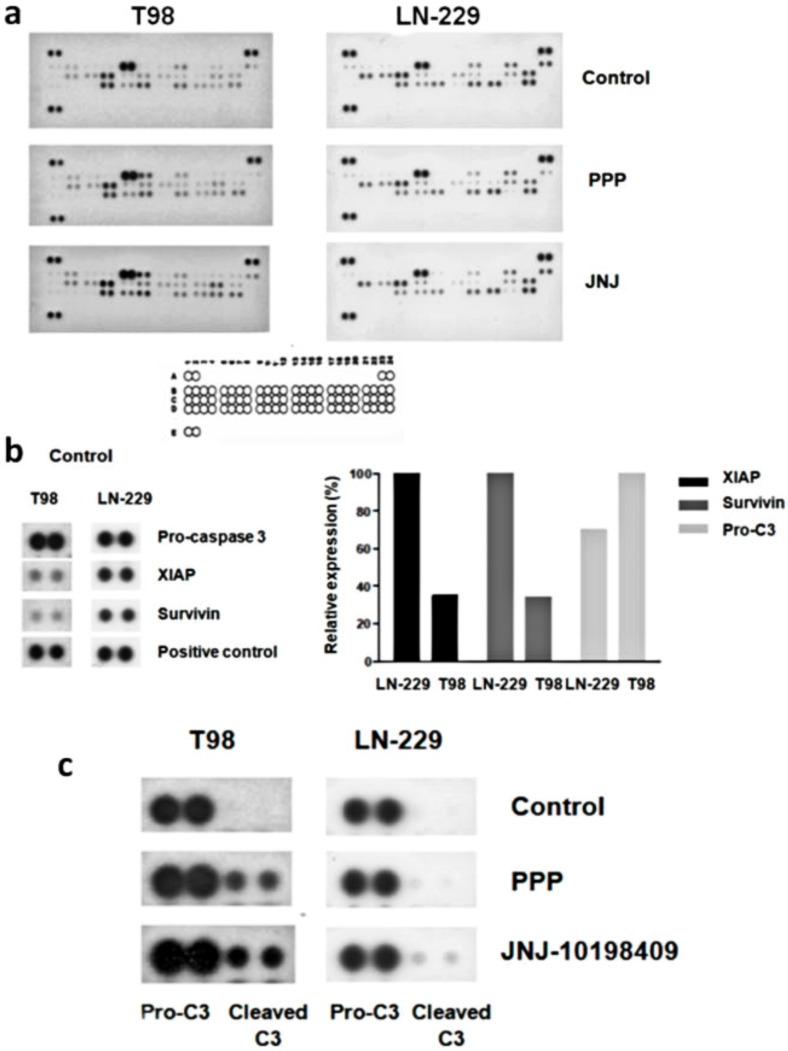
JNJ and PPP effects on the apoptotic proteome of GBM cells indicated by apoptosis arrays. (**a**) Effects on levels and phosphorylation status of 35 proteins related to apoptosis. T98 and LN-229 cells were treated with DMSO (Control), 500 nM PPP or 500 nM JNJ for 24 h and subjected to apoptosis arrays, as described in Materials and Methods. Legend: A1, A2, A23, A24, E1 and E2: Positive controls. B1 and B2: Bad. B3 and B4: Bax. B5 and B6: Bcl-2. B7 and B8: Bcl-x. B9 and B10: Pro-Caspase-3. B11 and B12: Processed caspase-3. B13 y B14: Catalase. B15 and B16: c-IAP-1. B17 and B18: c-IAP-2. B19 and B20: Claspin. B21 and B22: Clusterin. B23 and B24: Cytochrome c. C1 and C2: TRAIL R1/DR4. C3 and C4: TRAIL R2/DR5. C5 and C6: FADD. C7 and C8: Fas/TNFRSF6. C9 and C10: HIF-1α. C11 and C12: HO-1/HSP32. C13 and C14: HO-2. C15 and C16: HSP27. C17 and C18: HSP60. C19 and C20: HSP70. C21 and C22: HTRA2/Omi. C23 and C24: Livin. D1 and D2: PON2. D3 and D4: p21/CIP1/CDNK1A. D5 and D6: p27/Kip1. D7 and D8: phospho-p53 (S15). D9 and D10: phospho-p53 (S46). D11 and D12: phospho-p53 (S392). D13 and D14: phospho-Rad17 (S635). D15 and D16: SMAC/Diablo. D17 and D18: Survivin. D19 and D20: TNF R1/TNFRSF1A. D21 and D22: XIAP D23 and D24: Negative control. (**b**) Cropped and enlarged figures from Figure 7a, showing levels of the most relevant apoptotic proteins. Steady-state levels of pro-caspase-3, XIAP and survivin in T98 and LN-229 cells are shown. Positive control is indicated in the enlarged image (left panel). Graphic representation of relative levels of pro-caspase-3, XIAP and survivin expression in T98 and LN-229 cells (right panel). (**c**) Cropped and enlarged figures from Figure 7a, showing caspase-3 processing. Enlarged image of pro-caspase-3 and processed caspase-3 in T98 and LN-229 cells non-treated (Control), or treated with 500 nM PPP or 500 nM JNJ for 24 h.

We performed apoptosis arrays in T98 and LN-229 treated with JNJ or PPP in order to analyze activation status or steady-state levels of 35 different proteins involved in cell death mechanisms (Figure 7a). Interestingly enough, we found that pro-caspase 3 protein levels were much higher in T98 cells than in LN-229 cells (Figure 7b) and that both inhibitors increased pro-caspase 3 activation in T98 cells. However, only JNJ but not PPP was able to induce pro-caspase 3 activation in LN-229 cells (Figure 7c). In contrast, protein levels of antiapoptotic members such as XIAP and survivin were much higher in LN-229 cells than in T98 cells (Figure 7b). 

### 3.10. Combination of PDGFR and IGF-1R Inhibition

**Figure 8:**
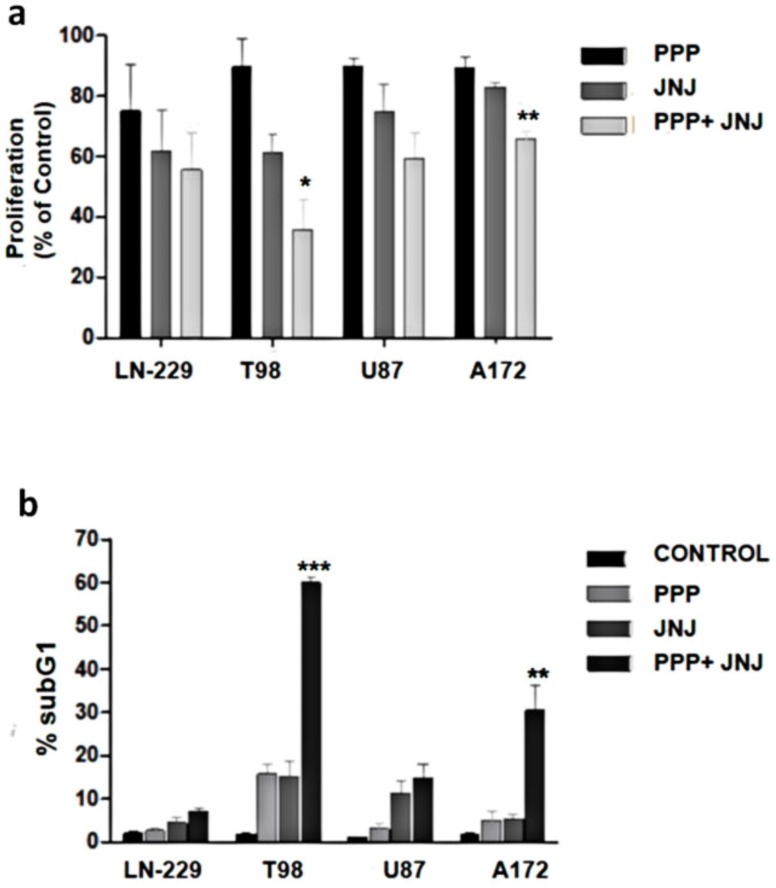
Combined effects of JNJ and PPP. (**a**) Effects on cell proliferation. GBM cells were seeded in 96-well plates, non-treated, or treated with 20 JNJ, 200 nm PPP or both for 72 h and cell proliferation was determined by MTT assays, as described in Materials and Methods, which is represented as the average of four independent experiments (*n* = 4) with six replicates in each experiment, and is referred to as percentage of control. Control bars have been omitted for being 100%. Error bars are the S.E.M. Difference between means was statistically significant * (*p* < 0.05), ** (*p* < 0.01). (**b**). Effects on cell death. GBM cells were non-treated (Control), or treated with 20 JNJ, 200 nm PPP or both for 72 h and subjected to cell cycle analyses. The percentage of cells in the SubG_1_ phase is represented as the average of six independent experiments (*n* = 6). Error bars are the S.E.M. Difference between means was statistically significant ** (*p* < 0.01), *** (*p* < 0.001).

We utilized JNJ and PPP at suboptimal concentrations to determine their combined effects. The effect of both inhibitors on cell proliferation was higher than either inhibitor alone, especially in T98 and A172 cells (Figure 8a). Likewise, cell cycle analyses revealed that the combination of both JNJ and PPP increased the percentage of cells in the SubG_1_ phase much more effectively than either inhibitor alone, especially in T98 and A172 cells (Figure 8b).

## 4. Discussion

In this report we have described the inhibition of PDGFR and IGFR-1 by JNJ and PPP, respectively. It has been shown that autocrine signaling through PDGFR contributes to glioblastoma proliferation, as PDGFR and its ligands are frequently coexpressed in glioblastoma cell lines and tumors [32]. In this report, we show that PDGFR activation, Akt and to a lesser extent, Erk 1/2 downstream signaling pathways (Figure 1) as well as proliferation of glioblastoma cell lines and primary cultures derived from tumors are efficiently inhibited by JNJ (Figure 2). Other PDGFR inhibitors such as Imatinib, Sumitinib, etc., which are undergoing evaluation in clinical studies, are actually multi-targeted kinase inhibitors. Therefore, we cannot rule out that some of the inhibitory effects observed by JNJ may be mediated by other targets. 

IGF-1R is a potential target for cancer treatment. In particular, signaling mediated by IGF-1R is essential for the development of glioblastoma [33,34]. The inhibitor PPP is able to impede IGF-1R activation, Akt and less importantly, Erk 1/2 phosphorylation (Figure 1), and proliferation of the cell lines and primary cultures utilized in this study (Figure 2). In contrast to PDGFR inhibitory agents, this inhibitor is very specific and does not inactivate the insulin receptor. Other groups have reported the inhibitory capacity of PPP in cell lines from different types of tumors [35,36,37,38,39,40], including glioblastoma cell lines [15]. 

Both inhibitors are able to arrest glioblastoma cell lines in G_2_/M, followed by an increase in the SubG_1_ phase. This effect is less dramatic in U87 cells, especially after PPP treatment. The fact that U87 cells have lower IGF-1R levels (Figure 1) may explain why they are less sensitive to this inhibitor. In fact, Akt and Erk 1/2 phosphorylation were barely diminished after PPP treatment in these cells (Figure 1c,d). The G_2_/M arrest provoked by JNJ and PPP treatment is further confirmed by cdc2 activation, as cdc2 Tyr 15 phosphorylation is decreased and Thr161 phosphorylation increased upon treatment. Moreover, steady-state levels of cyclin B_1_ protein also increased, which may indicate that cells are blocked in mitosis and cannot proceed to enter the next G_1_ phase. Furthermore, nuclei fragmentation and aggregation and micronuclei formation, which are characteristic of mitotic catastrophe, can be detected, whereas apoptosis-induced chromatin condensation could not be unequivocally observed (Figure S1). However, the increase in mitotic cells is higher upon PPP treatment than after JNJ treatment (Figure 4b), indicating that both mechanisms though similar have some differences. 

The dissimilarities between these two mechanisms were further investigated by analyzing the involvement of caspases in the cell death induction by either JNJ or PPP. PDGFR inhibition by JNJ induced a caspase-dependent cell death mechanism, which was especially evident in T98 cells (Figure 5) and confirmed by PARP cleavage and Annexin-V assays (Figure 6). However, cell death induction by IGF-1R inhibition after PPP treatment has a caspase-independent component, especially in LN-229 cells, where no PARP cleavage was observed and Annexin-V induction was less evident. It is interesting to note that the reduction of cell death achieved by the pan-caspase inhibitor in combination with PPP reaches the same levels in four cell lines, which is about 20% (Figure 5b). This indicates that PPP can trigger two cell death mechanisms: The first is caspase-dependent and has also been described in tumor cells of different origin [41,42], and the second is caspase-independent and it seems to be the only mechanism present in LN-229 cells. In order to find which other mechanism would explain cell death caused by PPP, we employed a battery of inhibitors in combination with PPP (Figure 6 and Figure S2). However, none of these inhibitors could clearly demonstrate which other pathway was involved. The caspase 8 inhibitor Ac-IETD-CHO did not have any effect (Figure 6A,B), which rules out the involvement of the extrinsic apoptotic pathway [22] as a mechanism of cell death.

Mitotic catastrophe is characterized as a poorly defined molecular pathway occurring during mitosis or as a result of mitotic failure, which precedes apoptosis, necrosis or senescence [43], with or without caspase intervention [44]. Cdc2 plays a role in mitotic catastrophe, which is inactivated by the kinase wee-1. The wee-1 inhibitor MK-1775 [23] could not abolish cell death induced by IGF-1R or PDGFR inhibition. Likewise, the microtubule-depolymerizing agent nocodazole arrested cells in mitosis, preventing the formation of mitotic spindles. The co-treatment of nocodazole with either PPP or JNJ could not prevent cell death, probably because cell death is initiated before mitotic spindle formation. In fact, it has been shown that JNJ induces mitotic arrest in glioblastoma-derived neuronal stem cells by inhibiting Plk1 phosphorylation [23]. However, even though PPP does not bind tubulin [45], we show in this report that this agent is able to induce a G_2_/M arrest, as it has been shown in multiple myeloma [35], classic Hodgkin lymphoma [46] and non-small lung cancer cells [47].

Autophagic cell death is a conserved catabolic process [48]. It has been proposed that there is extensive cross talk between autophagy and cell death. Autophagy can either protect cells from cell death induced by anti-tumor agents [49,50,51], or in other instances, it can enhance cell death [52,53]. Interestingly, cell death was induced by chloroquine treatment as a single agent in LN-229 cells, indicating a possible compensatory mechanism. However, autophagy inhibition could only partially prevent cell death induction by either PPP or JNJ in T98 cells. 

Necroptosis has been defined as a type of programmed necrosis [54]. The specific necroptosis inhibitor, necrostatin-1 [24], could not abolish PPP or JNJ-induced cell death, indicating that necroptosis is not involved in cell death mechanisms provoked by IGF-1R or PDGFR inhibition.

The results shown herein indicate JNJ-induced cell death has features of a canonical apoptotic caspase-dependent type of cell death. However, PPP-induced cell death has a caspase-independent non-classic component that could not be abolished using several drugs that target molecules involved in different types of cell death. Therefore, even though we have no clear evidence of the nature of this caspase-independent cell death mechanism, we presume that it is a necrotic, non-programmed type of death, probably resulting from damage of the mitotic machinery. In fact, it has been suggested that cell death in mitosis can be the result of mechanical damage, rather than programmed cell death [55]. The apoptosis arrays performed in LN-229 cells and T98 cells after JNJ or PPP treatment (Figure 7) revealed that LN-229 is less prone to undergo caspase-dependent cell death mechanisms than T98 cells, where procaspase-3 levels are higher and anti-apoptotic members such as XIAP and survivin are lower. Therefore, the T98 molecular context is more susceptible to an apoptotic cell death mechanism than the LN-229 molecular background, where other non-apoptotic caspase-independent mechanisms are more predisposed to be activated. Moreover, the effect of combining PPP and JNJ was much higher than the effect of each agent alone (Figure 8). This was especially evident in the amount of cell death in T98 cells (Figure 8b), which indicates that cell death mechanisms triggered by IGF-1R inhibition and PDGFR inhibition are cooperative, confirming similar results already shown in glioblastoma [56,57].

## 5. Conclusions

In this paper, we show that IGF-1R and PDGFR inhibition are powerful strategies for combating glioblastoma. We determined the effects of the PDGFR inhibitor JNJ-10198409 (JNJ) and the IGF-1R inhibitor picropodophyllin (PPP) in glioblastoma cellular models. Both inhibitors inhibited cell proliferation and induced a G_2_/M arrest with a high proportion of cells accumulated in mitosis, followed by an increase in the SubG_1_ phase, indicative of cell death. JNJ-induced cell death was caspase-dependent, Annexin-V positive and produced PARP cleavage, especially in T98 cells, indicating that cell death induced by PDGFR inhibition follows a canonical apoptotic mechanism. However, PPP-induced cell death was abolished by caspase inhibitors only to a certain extent in all cell lines except LN-229 cells, revealing a caspase-independent mechanism. Several inhibitors targeted against different cell death pathways could not eliminate this caspase-independent component, which presumably is a non-programmed necrotic mechanism due to mechanical failure after the mitotic arrest. Apoptotic arrays indicated that procaspase 3 levels were induced by both agents in T98 cells and only by JNJ in LN229-cells. Moreover, XIAP and survivin levels were much higher in LN-229 cells than in T98 cells, suggesting that LN-229 cells are more prone to undergo caspase-independent cell death mechanisms. Furthermore, the combination of JNJ and PPP was cooperative and more efficacious than each treatment alone. Therefore, the combination of agents that inhibit both the IGF-1R and the PDGFR pathways could contribute to improve the treatment of glioblastoma patients.

## Supplementary Materials

The following are available online at http://www.mdpi.com/2073-4409/7/9/131/s1, Figure S1: PPP effect on chromatin integrity, Figure S2: Caspase-independent mechanisms.
