# Supplementary material for: PDGFR and IGF-1R Inhibitors Induce a G2/M Arrest and Subsequent Cell Death in Human Glioblastoma Cell Lines
Source: Cells. 2018 Sep 6;7(9):131. doi: 10.3390/cells7090131 (PMC6162497; doi:10.3390/cells7090131)
Supplement: Supplementary file 1 [file cells-07-00131-s001.pdf]

Supplementary Materials:

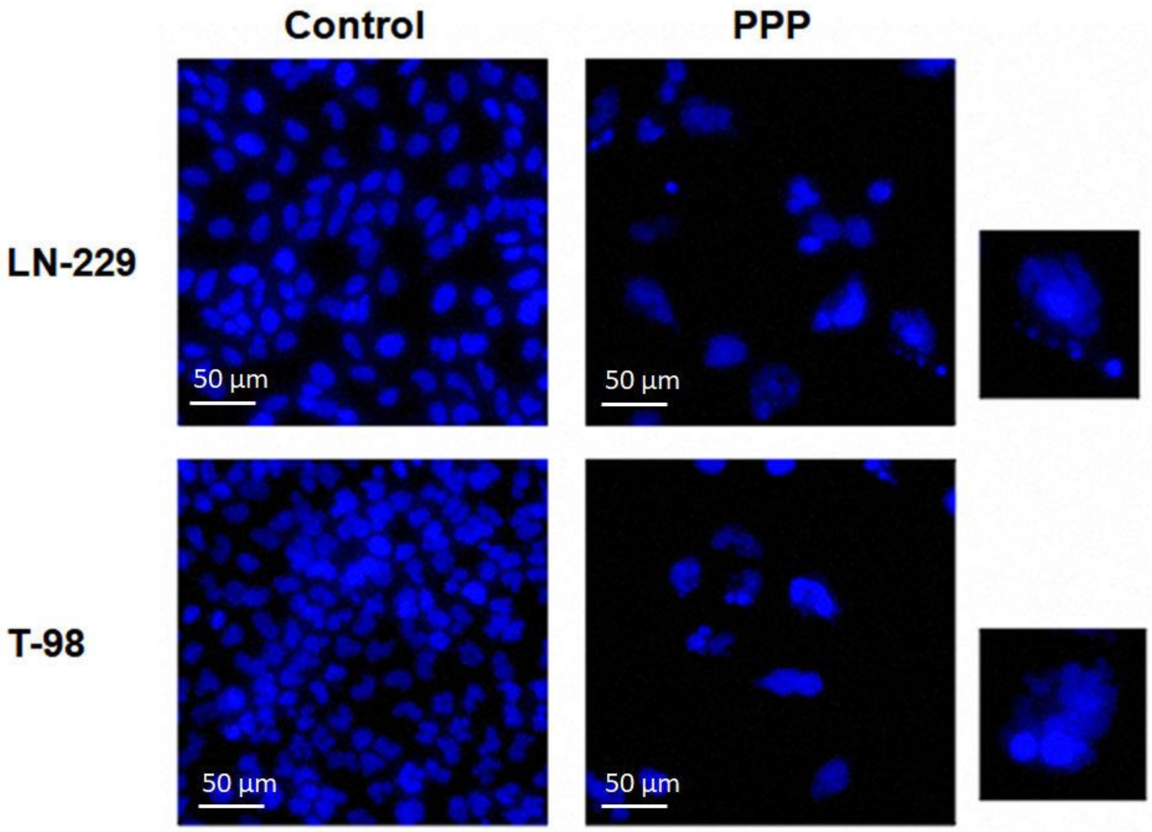

Figure S1. PPP effect on chromatin integrity. GBM cells were plated, treated with 500 nM PPP for 72 h and stained with Hoechst 33342 as described in Materials and Methods.

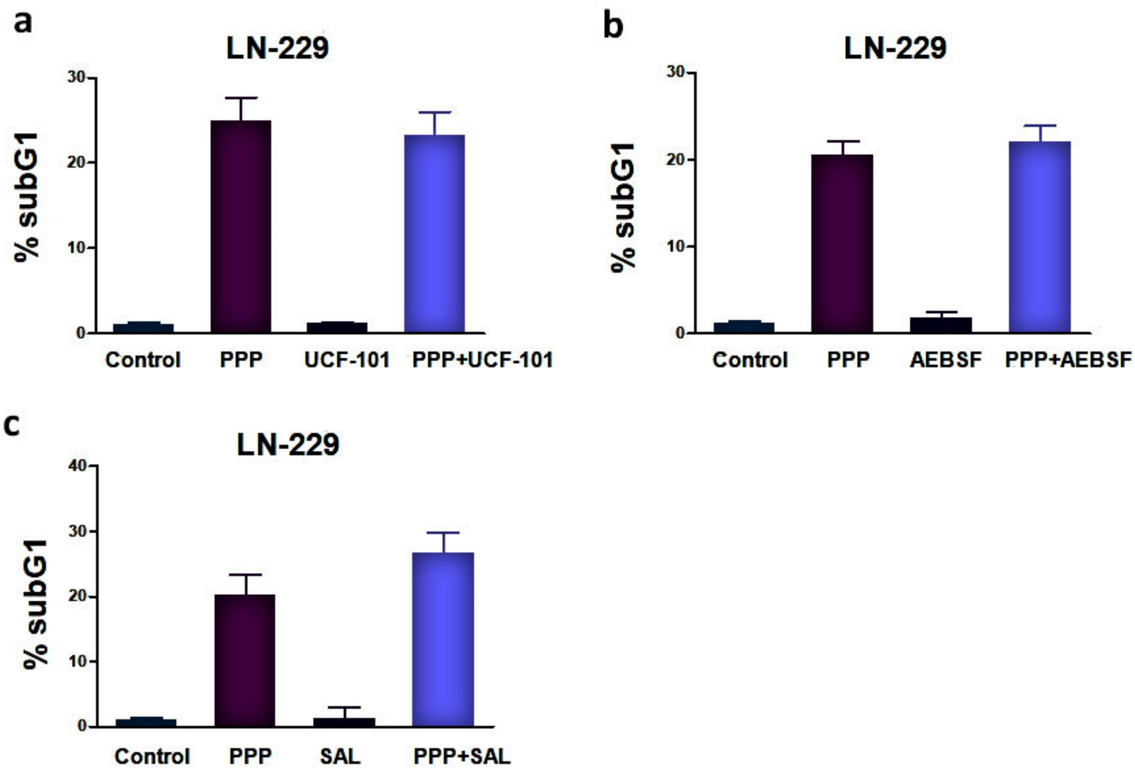

Figure S2. Caspase-independent mechanisms. LN-229 cells were seeded and treated with 500 nM PPP for 72 h in the presence or absence of (**A**) 10 mM of the Omi inhibitor Ucf-1, (**B**) 100 mM of the Serine protease inhibitor AEBSF or (**C**) 100 mM of the eIF2α inhibitor Salubrinal. Cell cycle analyses were performed by flow cytometry, as described in Materials and Methods and the percentage of cells in the SubG$_1$ phase is represented. Graphs represent the average of at least three independent experiments. Error bars are the S.E.M (n = 4).
